# Comprehensive Analysis of circRNAs, miRNAs, and mRNAs Expression Profiles and ceRNA Networks in Decidua of Unexplained Recurrent Spontaneous Abortion

**DOI:** 10.3389/fgene.2022.858641

**Published:** 2022-05-31

**Authors:** Xiaohua Liu, Jiabao Wu, Hua Nie, Xiaoli Zhu, Ge Song, Lu Han, Weibing Qin

**Affiliations:** ^1^ NHC Key Laboratory of Male Reproduction and Genetics, Guangzhou, China; ^2^ Department of Center Laboratory, Guangdong Provincial Reproductive Science Institute (Guangdong Provincial Fertility Hospital), Guangzhou, China; ^3^ Reproductive Medicine Center, Guangdong Provincial Reproductive Science Institute (Guangdong Provincial Fertility Hospital), Guangzhou, China

**Keywords:** the unexplained recurrent spontaneous abortion, circRNA, RNA sequencing, CeRNA networks, protein-protein interaction

## Abstract

The diagnosis and treatment of unexplained recurrent spontaneous abortion (URSA) are subject to debate, because the exact underlying mechanisms remain unclear. To address this issue, we elucidated the expression profiles of dysregulated circRNAs, miRNAs, and mRNAs and constructed circRNA-associated competitive endogenous RNA (ceRNA) networks by comparing the decidua of URSA with that of normal early pregnancy (NEP) using RNA-sequencing. In total, 550 mRNAs, 88 miRNAs, and 139 circRNAs were differentially expressed (DE) in decidua of URSA. Functional annotation revealed that DE mRNAs as well as potential target genes of DE miRNAs and DE circRNAs are mainly involved in immunologic function, such as antigen processing and presentation, allograft rejection, and T cell receptor signaling pathway. In addition, the top hub genes, including *CCL4, DDX58, CXCL10, CXCL9, MX1, CD44, RPS2, SOCS3, RPS3A,* and *CXCL11*, were identified. The mRNAs involved in ceRNA network were enriched in complement and coagulation cascades and protein processing in the endoplasmic reticulum. We found that circRNAs in the ceRNA network, which acted as decoys for hsa-miR-204-5p, were positively correlated with *MFGE8* expression. Collectively, the results demonstrated that circRNAs, miRNAs, and mRNAs were aberrantly expressed in the decidua of patients with URSA and played a potential role in the development of URSA. Thus, the establishment of the ceRNA network may profoundly affect the diagnosis and therapy of URSA in the future.

## Introduction

Recurrent spontaneous abortion (RSA), which refers to three or more spontaneous abortions with a single spouse, occurs in 1–5% of women of childbearing age and exerts a negative impact on human reproductive health (Medicine, 2008). Although the etiology of RSA has not yet been completely clarified owing to its complicated molecular and cellular regulation, known causes include chromosomal translocations, uterine abnormalities, thrombophilias, endocrine defects, autoimmune diseases, and infectious agents (Srinivas et al., 2006; Cohn et al., 2010; Daher et al., 2012; Rull et al., 2012; Asadpor et al., 2013; Pluchino et al., 2014; Dean et al., 2019). However, the cause and pathogeneses of approximately 40–50% of RSA cases remain unknown (Stephenson, 1996; Weimar et al., 2012); these are termed unexplained RSA (URSA). URSA poses a major obstacle for many couples who desire to conceive a child. Recent studies have shown a correlation between noncoding RNAs (ncRNAs) and the pathogenesis of URSA (Wang et al., 2012; Dong et al., 2014).

Circular RNAs (circRNAs) a class of ncRNAs, are characterized by a covalent bond linking the 3' and 5' ends generated by back splicing. With the advent of high-throughput RNA sequencing technology, circRNAs that were considered as nonfunctional (Capel et al., 1993), were found to be involved in many diseases, such as atherosclerosis, neurological diseases, diabetes, and cancer, all of which involve processes similar to those of embryo implantation (Chen L. et al., 2015; Qu et al., 2015). In recent years, some studies have investigated the role of circRNAs in URSA. Li et al. compared the decidua tissues of the early recurrent miscarriage group with those of the control group using circRNA microarray analysis and identified differentially expressed (DE) circRNAs (Li C. et al., 2020). Another study used an RSA mouse model to demonstrate a molecular mechanism involving migration and invasion of circ-ZUFSP regulating trophoblast cells (Li Z. et al., 2020). This provides a new indicator for early diagnosis and potential treatment of RSA. However, a comprehensive understanding of the processes involved in URSA is lacking, and the potential role of circRNAs, miRNAs, and mRNAs in URSA remains unresolved.

The purpose of the current study was to gain an overview of the expression profiles of circRNAs, miRNAs, and mRNAs in URSA via RNA sequencing. In addition, because the role of circRNAs as miRNA sponges that regulate placental behavior has been confirmed (Ou et al., 2019), we constructed a circRNA-miRNA-mRNA interaction network to investigate the potential functions of these DE circRNAs. Our findings provide new evidence that may help better understand the molecular mechanisms underlying the role of circRNAs, miRNAs, and mRNAs in the pathogenesis of URSA.

## Materials and Methods

### Sample Collection

All participants were recruited from the Outpatient Department of Guangdong Provincial Fertility Hospital (Guangzhou, China), during the period between June 2016 and December 2019. Patients included in the current study provided written consent before surgery, and the Ethical Committee of the Guangdong Provincial Fertility Hospital approved the study protocol. The decidua tissues were obtained from 9 women (age: 27 ± 6.38 years and gestational age: 8.36 ± 0.36 weeks) *via* dilation and curettage following spontaneous abortion. Normal decidua tissues were obtained from 9 women (age: 26.67 ± 3.64 years and gestational age: 7.88 ± 0.8 weeks) during early pregnancy (6–12 weeks) *via* dilatation and curettage after the termination of pregnancy. The clinical data are presented in Table 1. Inclusion criteria for URSA were three or more consecutive spontaneous abortions and no history of successful pregnancies. Exclusion criteria for URSA were infections, endocrine or metabolic disorders, anatomic abnormalities, autoimmune diseases, paternal or maternal chromosomal abnormalities, and abnormal karyotyping of the miscarriage product. Inclusion criteria for the normal controls were 1) elective termination of normal early pregnancy, 2) at least one live birth, and 3) no history of miscarriage, preeclampsia, ectopic pregnancy, preterm delivery, and systemic diseases. After dilation and curettage, decidua tissues were immediately separated from the products of conception, washed thoroughly with sterile normal saline, and stored in liquid nitrogen for future use.

**TABLE 1:** Clinical characteristics of the participants.

Characteristic	URSA (*n* = 9)	NEP (*n* = 9)	*P*
Age (years)	27 ± 6.38	26.67 ± 3.64	0.894
Gestational age (week)	8.36 ± 0.36	7.88 ± 0.8	0.267
BMI	21.89 ± 1.74	22.04 ± 0.98	0.327

Patient age, gestational ageand BMI at blood sampling in URSA and NEP groups are indicated as mean and standard deviation.Significant difference between groups were analyzed by Student’s *t* test. *p <* 0.05 was considered statistically significant.

URSA, unexplained recurrent spontaneous abortion; NEP, normal early pregnancy; BMI, body mass index.

### RNA Library Preparation and Sequencing

A total amount of 5 μg RNA per sample was used as input material for the RNA sample preparations. Firstly, ribosomal RNA was removed by Epicentre Ribo-zero™ rRNA Removal Kit (Epicentre, United States), and rRNA free residue was cleaned up by ethanol precipitation. The sequencing libraries were generated by NEBNext® Ultra™ Directional RNA Library Prep Kit for Illumina® (NEB, United States) following manufacturer’s recommendations. In addition, A total amount of 3 μg total RNA per sample was used as input material for the small RNA library. Sequencing libraries were generated using NEBNext ® Multiplex Small RNA Library Prep Set for Illumina ® (NEB, United States) following manufacturer’s recommendations and index codes were added to attribute sequences to each sample. The clustering of the index coded samples was performed on a cBot Cluster Generation System using TruSeq SR Cluster Kit v3 cBot HS (Illumina) according to the manufacturer’s instructions. After cluster generation, the library preparations were sequenced on an Illumina Hiseq 2500/2000 platform and 50bp single-end reads were generated. The raw sequencing data presented in the study has been deposited in the Sequence Read Archive (SRA) database (SRA: PRJNA819210, PRJNA819201).

### Protein-Protein Interaction Network Construction, Hub Gene Selection, and Module Analysis

The protein level interactions of DE mRNAs were analyzed using the Search Tool for the Retrieval of Interacting Genes/Proteins (STRING) database (https://string-db.org/cgi/input.pl). Interactions with a combined score ≥0.4 were considered statistically significant. Cytoscape software (version 3.7) was applied to visualize the PPI network. The proteins in the central node (hub proteins) that were highly connected to a series of genes in the network were considered most closely associated with pathogenesis. The top 10 hub genes, ranked by degree of connectivity, were identified using the cytoHubba APP plug-in of Cytoscape. Significant modules in the PPI networks were determined using the Molecular Complex Detection (MCODE) APP (v1.5.1) plug-in of Cytoscape.

### Real-Time Quantitative PCR (RT-qPCR) Validation

Total RNAs were extracted using a Qiagen RNeasy mini kit (Qiagen, Hilden, Germany) and reverse transcribed using SuperScript III First-Strand Synthesis system (Life Technologies, Carlsbad, CA, USA). Real-time quantitative PCR was carried out using TB Green Premix EX Taq II (Takara, Beijing, China) and analyzed using the AB Step One Plus System (Applied Biosystems, AB, United States). Convergent primers were designed for linear mRNA production while divergent primers targeting the predicted back-spliced region were designed for the circular form. The specific quantitative primers designed using oligo7 software are listed in Supplementary Table S1. Primers of *β*-actin (for circRNA and mRNA) and U6 (for miRNA) were designed as an endogenous control. Each experiment was performed in triplicate.

### GO and KEGG Analyses and GSEA

GO, which describes genes from any organism (http://www.geneontology.org), covering the domains of Biological Process (BP), Cellular Component (CC), and Molecular Function (MF), was used. KEGG pathway analysis (http://www.genome.jp/kegg) was used to analyze the functions of mapped genes. Fisher’s exact test, the chi-square test, and the false discovery rate (FDR) were used for level of significance detection, where the *p*-value denoted the significance of the GO term and pathways correlated with the condition. A small FDR indicated a small error in judging the *p*-value. Gene Set Enrichment Analysis (GSEA) was performed using GSEA-P software to evaluate the RNA-Seq data at the gene level, with gene sets from the Molecular Signatures Database (MSigDB).

### Construction of a circRNA-miRNA-mRNA Network

The expression levels of circRNAs, miRNAs, and mRNAs that showed significant differences between URSA and NEP were analyzed. We used miRanda (http://www.microrna.org/microrna/) to predict miRNA binding seed sequence sites, where overlapping of the same miRNA binding sites on both circRNAs and mRNAs indicated circRNA-miRNA-mRNA interactions. Finally, we constructed the network using Cytoscape for Windows (National Institute of General Medical Sciences) software (Supplementary Figure S1).

### Western Blot Analysis

Briefly, whole protein samples were extracted, and their concentrations were measured using a Pierce BCA Protein Assay Kit (Thermo Fisher Scientific), following which 20 μg of protein was loaded and separated on a 10% sodium dodecyl sulfate (SDS) polyacrylamide gel. The membranes were incubated overnight at 4°C with specific primary antibodies MFGE8 (Abcam, dilution in 1:500) and GAPDH (Proteintech, dilution in 1:1000), followed by incubation with goat anti-rabbit IgG conjugated to horseradish peroxidase (Santa Cruz, dilution in 1:5000) for 1 h at room temperature. The membranes were then visualized *via* a film following incubation with ECL solution (Cell Signaling Technology).

### Statistical Analysis

Statistical analyses were performed using SPSS 20.0 software. All data were expressed as mean ± SD. Statistical significance was set at *p* < 0.05. The Student’s *t* test was used to compare RT-qPCR results.

## Results

### Identification of DE circRNAs, miRNAs, and mRNAs

**FIGURE 1:**
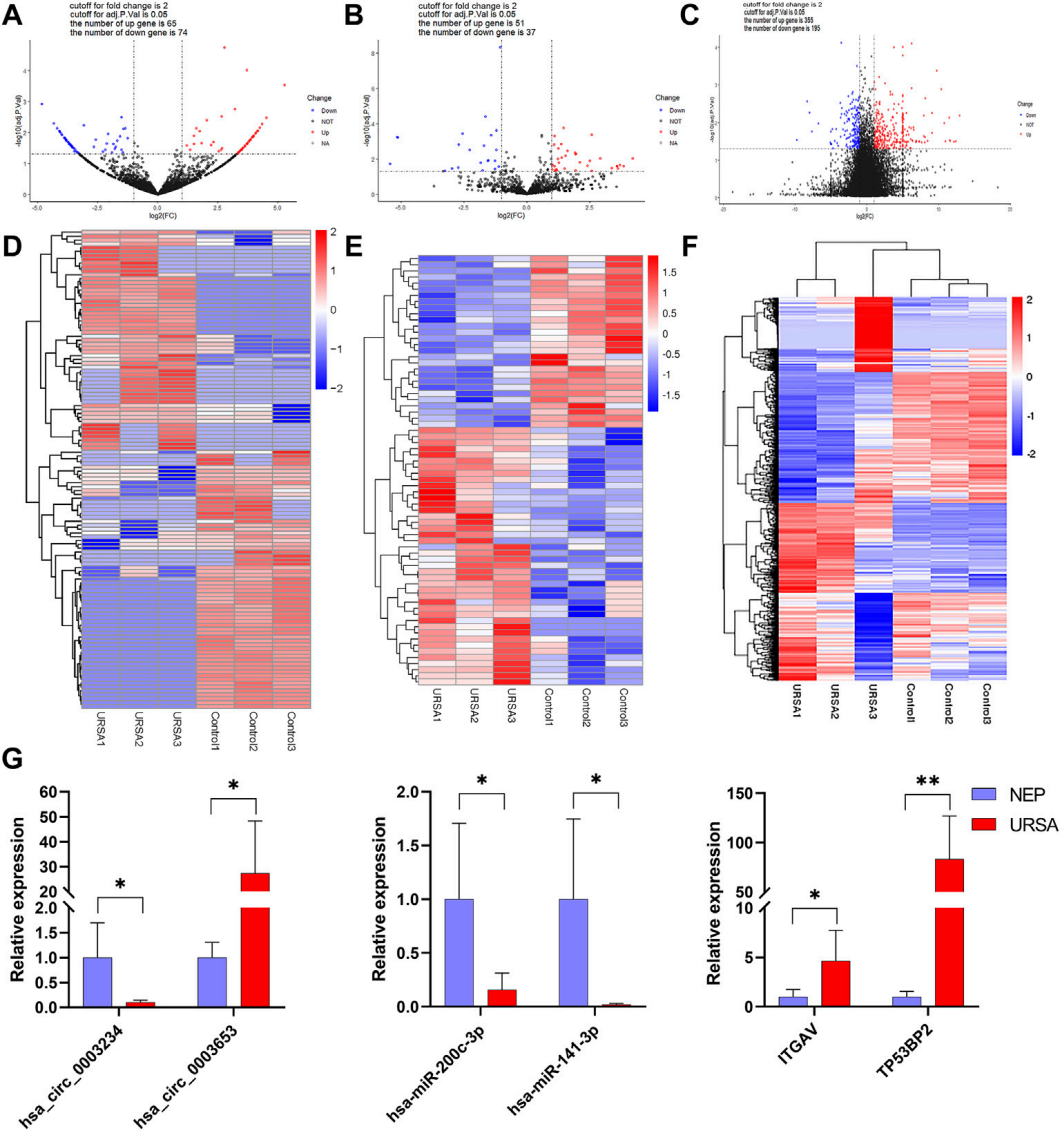
The expression profiles of global circRNAs, miRNAs and mRNAs in decidua of URSA and NEP. **(A–C)** Volcano plots of differentially expressed circRNAs **(A)**, miRNAs **(B)**, and mRNAs **(C)**. Red dots indicate upregulated and blue dots indicate downregulated RNAs. Heat map of differentially expressed circRNAs **(D)** miRNAs **(E)** and mRNAs **(F)** generated with RNA-seq in decidua of URSA patients and NEP. **(G)** The relative expression levels of six RNAs in URSA and NEP verified using RT-qPCR. URSA, unexplained recurrent spontaneous abortion; NEP, normal early pregnancy, *p < 0.05, **p < 0.01.

The expression of circRNAs, miRNAs, and mRNAs in the URSA and NEP control groups were analyzed using RNA sequencing. After processing the raw data, we identified 5126 circRNAs, 1540 miRNAs, and 16065 mRNAs that were aberrantly expressed in URSA. Subsequently, we used *p* < 0.05 as the selection criteria and determined that 139 circRNAs were DE in the decidua of RSA patients, of which 65 DE circRNAs were upregulated and 74 DE circRNAs were downregulated (Figure 1A; Table 2). Further, differences in miRNA profiles of URSA and control groups in response to changes in the expression profiles of circRNAs were investigated. Of these, 88 were DE miRNAs, including 51 upregulated DE miRNAs and 37 downregulated DE miRNAs (Figure 1B; Table 3). Besides these, 355 and 195 DE mRNAs were upregulated and downregulated, respectively (Figure 1C; Table 4). Results of the cluster analyses of the expression levels of DE circRNAs, microRNAs, and mRNAs are displayed in Figures 1D–F. Six DE RNAs (hsa_circ_0003653 and hsa_circ_0003234, hsa-miR-200c-3p, hsa-miR-141-3p, ITGVA, TP53BP2) were further verified by RT-qPCR, showing a significant difference between URSA and NEP, which was consistent with the RNA-sequencing results (Figure 1G).

**TABLE 2:** Top 10 differentially expressed circRNAs in decidua of URSA patients.

circRNA	Regulation	Log_2_ FC	*p* value
hsa_circ_0003653	Up	2.7813	1.86E-05
novel_circ_0008332	Up	3.6976	9.85E-05
hsa_circ_0005835	Up	5.2764	0.000287
novel_circ_0005534	Up	3.2178	0.001754
hsa_circ_0008421	Up	2.6548	0.003042
hsa_circ_0003234	Down	−4.8209	0.001219
hsa_circ_0006654	Down	−1.5197	0.003254
hsa_circ_0000504	Down	−3.0826	0.005885
hsa_circ_0001358	Down	−1.3512	0.007381
novel_circ_0001987	Down	−1.4358	0.007492

**TABLE 3:** Top 10 differentially expressed microRNAs in decidua of URSA patients.

miRNA	Regulation	Log_2_ FC	*p* value
hsa-miR-548ba	Up	1.721	8.00E-05
hsa-miR-132-5p	Up	1.2477	0.00015105
hsa-miR-221-5p	Up	0.98587	0.00080257
hsa-miR-29c-5p	Up	1.1415	0.0010109
hsa-miR-95-3p	Up	1.2433	0.0014506
hsa-miR-200c-3p	Down	−0.99993	7.72E-06
hsa-miR-1307-5p	Down	−1.3846	4.07E-05
hsa-miR-4521	Down	−1.7329	6.17E-05
hsa-miR-1246	Down	−1.6176	0.00036373
hsa-miR-141-3p	Down	−1.0639	0.00036617

**TABLE 4:** Top 10 differentially expressed mRNAs in decidua of URSA patients.

mRNA	Regulation	Log_2_ FC	*p* value
TP53BP2	Up	6.261922625	7.95E-05
ITGAV	Up	infinity	9.77E-05
ZNF331	Up	3.777094197	9.90E-05
DDX3X	Up	infinity	0.000163
IST1	Up	0.622679087	0.000173
CADPS2	Down	−3.5695	7.59E-05
ZNF250	Down	−1.37865	0.000314288
MINA	Down	−0.26239	0.000347628
EEF2K	Down	−0.84928	0.000425782
GCAT	Down	−0.73805	0.000532758

### GO and KEGG Pathway Analysis of DE mRNA Combined With GSEA

**FIGURE 2:**
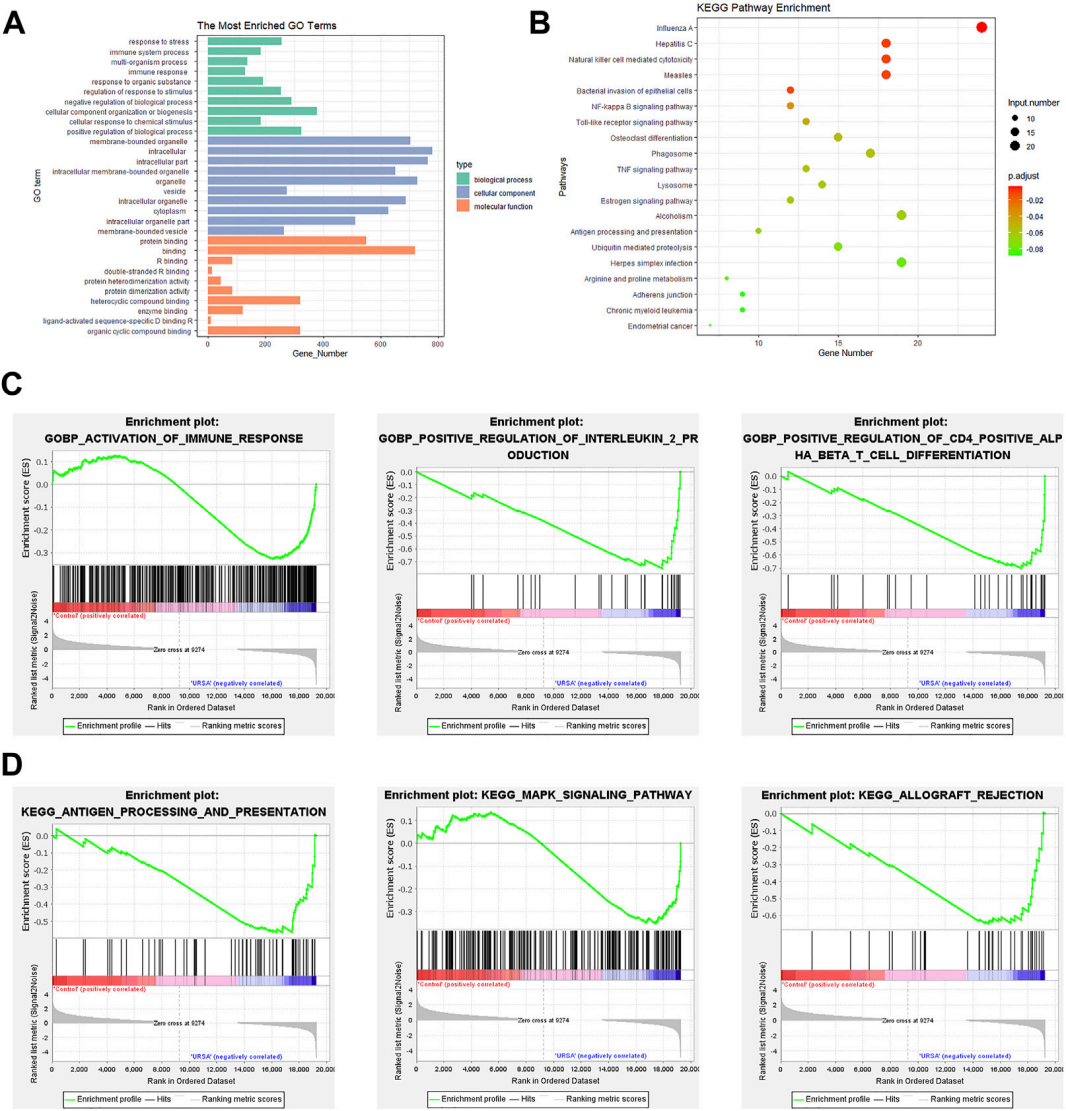
GO and KEGG pathway enrichment analyses and GSEA. GO analysis **(A)** and KEGG **(B)** of dysregulated mRNAs. **(C)** GSEA showed DE mRNAs involved in the BP GO annotations. **(D)** GSEA showed DE mRNAs in KEGG.

GO, and KEGG analyses were conducted to predict the potential functions of the DE mRNAs. DE mRNAs in the BP group were mainly enriched in immune response, immune system process, and regulation of response to stimuli (Figure 2A). Furthermore, evaluation of RNA-seq data at the genome-wide level via GSEA confirmed and supplemented the GO results. Using BP GO annotations, GSEA showed that the activation of immune response, positive regulation of interleukin 2 production and positive regulation of CD4 positive alpha-beta T cell differentiation were upregulated in the decidua of URSA (Figure 2C). To determine the signaling pathways associated with DE-mRNAs, KEGG pathway analysis was performed. The DE mRNAs were primarily enriched in antigen processing and presentation, Toll-like receptor signaling pathway, and Natural killer cell mediated cytotoxicity (Figure 2B). GSEA results annotated via the KEGG pathway further showed that gene sets regulating antigen processing and presentation, the MAPK signaling pathway, and allograft rejection were upregulated in the decidua of URSA (Figure 2D).

### PPI Analysis of DE mRNAs

**FIGURE 3:**
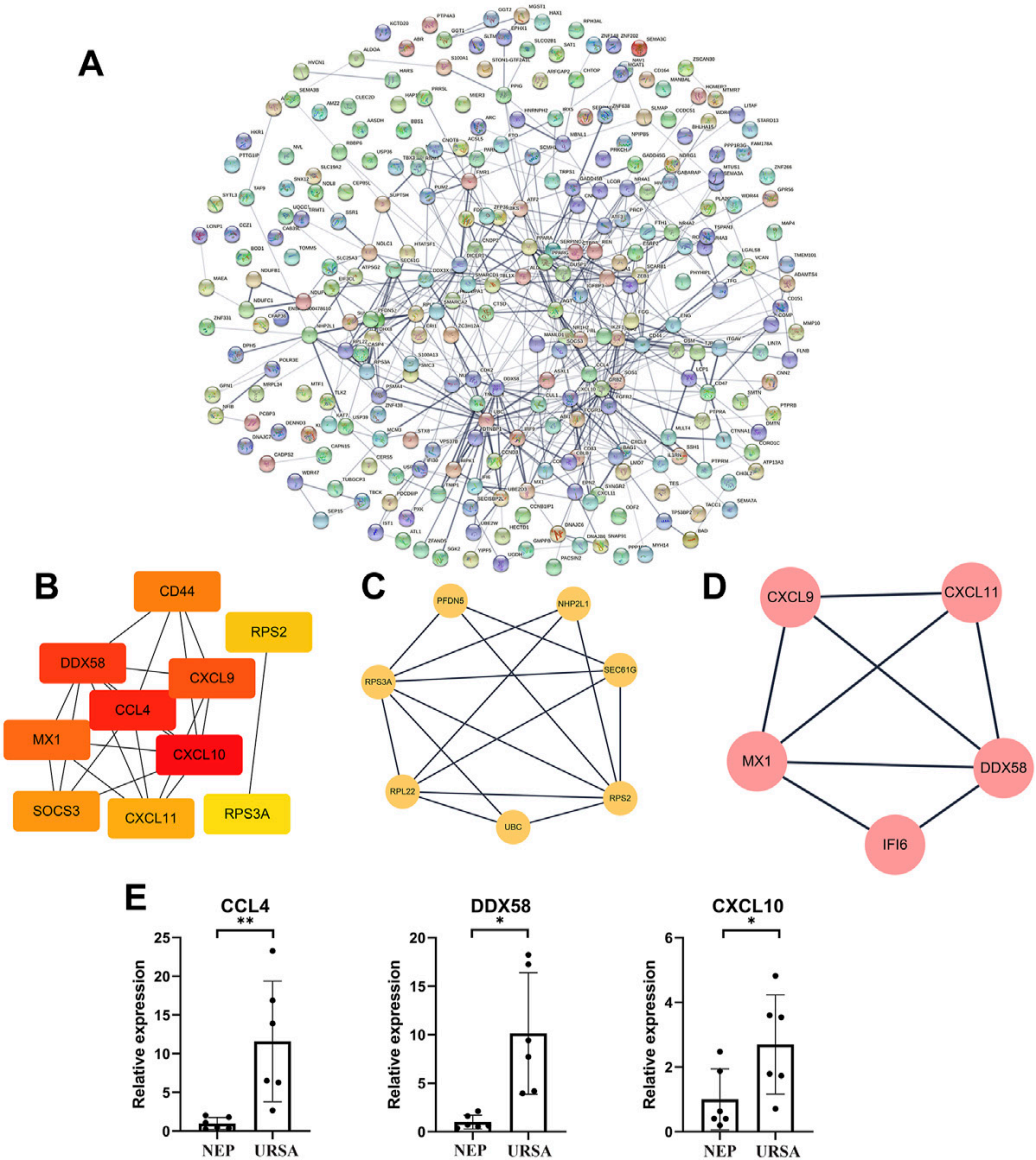
PPI network analysis of DE mRNAs and validation of mRNA-Seq data. **(A)** The PPI network of DE mRNAs. **(B)** The subnet involving the top 10 DEGs (hub genes) with high connectivity degrees obtained from the PPI network. **(C,D)** The top 2 significant modules were obtained from the PPI network. **(E)** The relative expression levels of three mRNAs in the PPI network were validated by RT-qPCR. *p < 0.05, **p < 0.01.

To further analyze the functions of DE mRNAs at the protein level and also to explore the core mRNAs involved in the cellular processes of URSA, we used the STRING database to screen for functional genes and constructed an intuitive network for the annotation of PPIs (Figure 3A). The core mRNAs (known as hub genes) that corresponded to the DEGs included *CCL4*, *DDX58*, *CXCL10, CXCL9*, *MX1, CD44*, *RPS2, SOCS3*, *RPS3A,* and *CXCL11* (Figure 3B). Module analysis revealed two closely connected regions with the highest scores in the PPI network (Figures 3C,D). *CCL4*, *DDX58,* and *CXCL10* in the PPI network were upregulated in decidua of patients with URSA (Figure 3E).

### Distinct circRNA and miRNA Expression Profiles in Decidua Tissues of Patients With URSA

**FIGURE 4:**
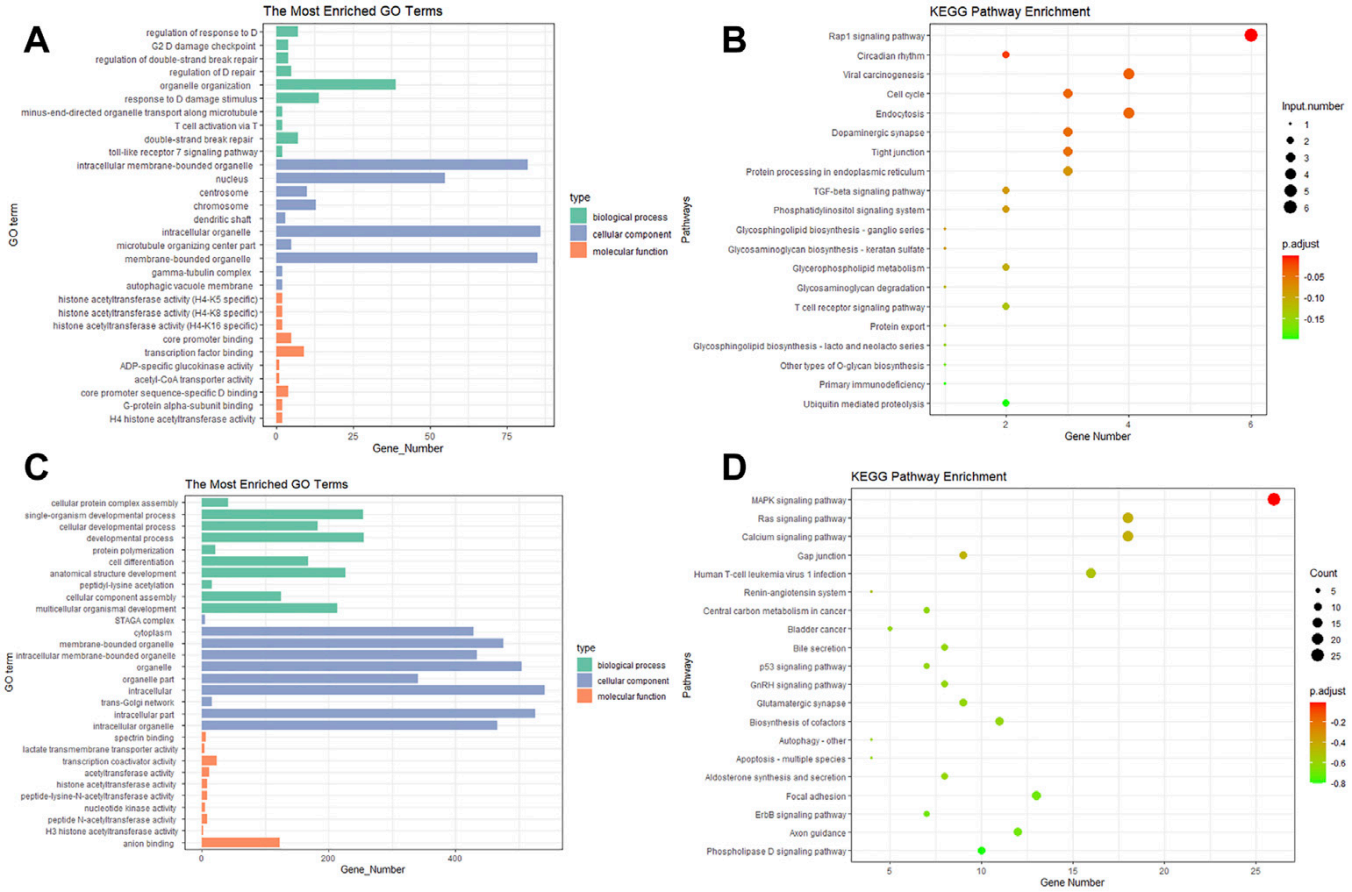
Gene ontology and pathway analysis of the potential target genes (PTGs) of DE miRNAs and circRNAs. **(A,C)** GO analysis of miRNA and circRNA, respectively. **(B,D)** KEGG of miRNA and circRNA, respectively.

GO analysis showed that the target genes of DE circRNAs in the biological processes mainly regulated response to DNA damage stimuli, T cell activation via T cell receptor contact with antigen bound to MHC molecules on antigen presenting cells, and the Toll-like receptor 7 signaling pathway (Figure 4A). The DE miRNAs were significantly enriched in cellular developmental processes, cellular component assembly, and developmental processes (Figure 4C).

KEGG pathway enrichment analysis revealed that the DE circRNAs were mainly enriched in the Rap1 signaling pathway, the TGF-beta signaling pathway, and the tight junction and T cell receptor signaling pathway (Figure 4B). The pathways enriched in miRNA included the MAPK signaling pathway, the Ras signaling pathway, and gap junction and focal adhesion (Figure 4D).

### Construction of circRNA-miRNA-mRNA ceRNA Network

**FIGURE 5:**
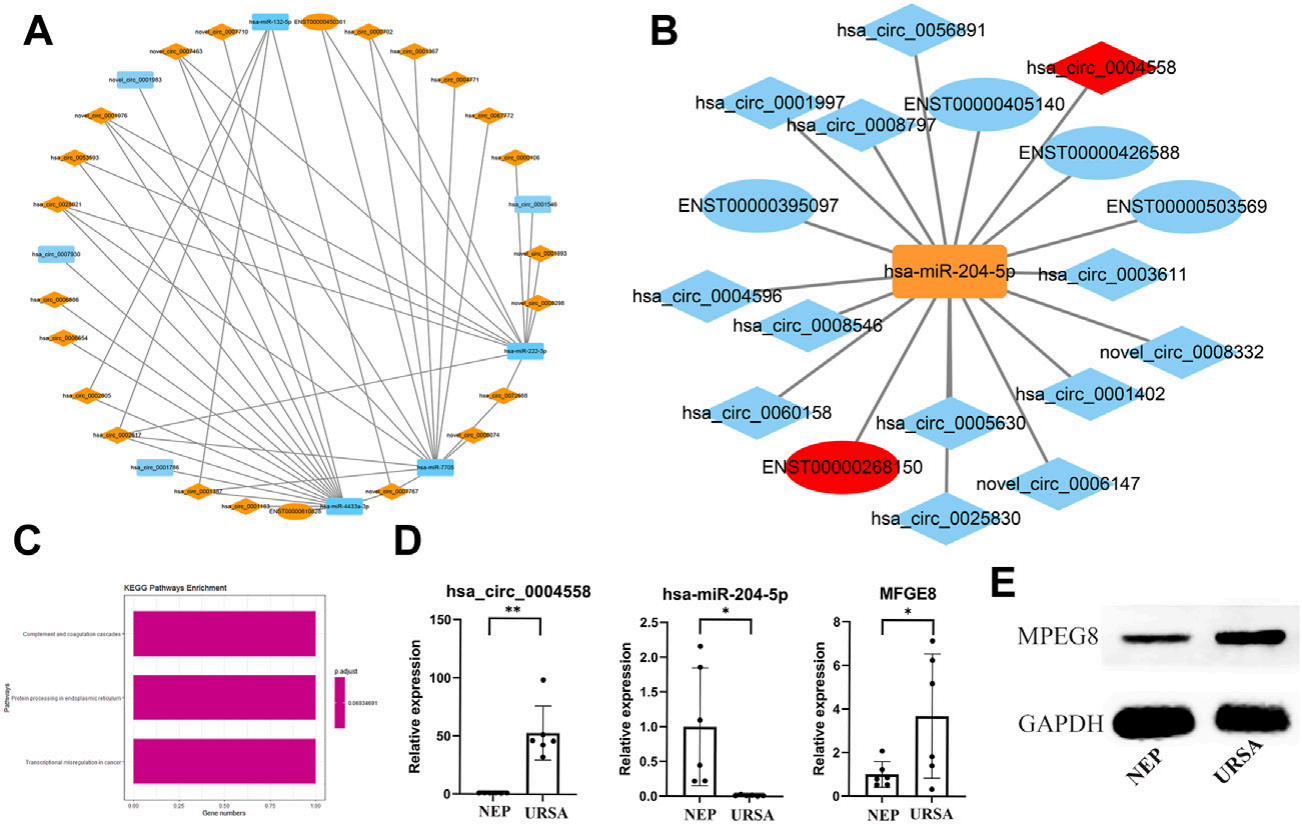
Establishment of a ceRNA network and validation of the expression of has-miR-204-5p and MFGE8. **(A)** circRNA (down in URSA)-miRNA (up in URSA)-mRNA (down in URSA).**(B)** circRNA (up in URSA)-miRNA (down in URSA)-mRNA (up in URSA), RNAs marked in red are most likely to participate in the pathogenesis of URSA. **(C)** The KEGG pathway of mRNA involved in ceRNA. **(D)** The expression of has-miR-204-5p/MFGE8 in decidua of URSA and NEP by RT-qPCR. **(E)** The expression of MFGE8 in decidua of URSA and NEP by western blot analysis. The rectangles indicate miRNAs, ellipses represent mRNAs, and diamonds represent circRNAs, *p < 0.05, **p < 0.01.

To identify the ceRNA network in URSA, we extracted miRNAs, mRNAs, and circRNAs that were abnormally expressed in the triple network and constructed a network based on the ceRNA hypothesis using Cytoscape (Figures 5A,B). The associated mRNA correlated pathways were complement and coagulation cascades, protein processing in the endoplasmic reticulum, and transcriptional misregulation in cancer (Figure 5C). We selectively analyzed pairs in which the circRNAs, miRNAs, and target genes were possibly associated with URSA. A subnetwork consisting of circRNAs (hsa_circ_0004558, novel_circ_0006147, hsa_circ_0008546, etc.) included those acting as ceRNAs of hsa-miR-204-5p targeting *MFGE8* (Figure 5B). Subsequently, hsa-miR-204-5p and *MFGE8* were selected for RT-qPCR validation, which revealed that the expression trends and amplitudes were consistent with those of RNA-seq (Figure 5D). To build on these observations, we further investigated MFGE8 protein expression in the decidua of URSA, and the result was in concordance with those of RT-qPCR (Figure 5E). Additional results are listed in Supplementary Table S2.

## Discussion

The current study revealed significant differences between the RNA expression profiles in the decidua tissues of URSA and NEP groups. The pathways of DE mRNAs as well as the potential target genes of DE miRNAs, and DE circRNAs were mainly involved in antigen processing and presentation, allograft rejection, the T cell receptor signaling pathway, and junction related pathways. A ceRNA network was constructed, which revealed that the pathways associated with the target mRNAs were complement and coagulation cascades and protein processing in the endoplasmic reticulum. Additionally, strict limitative requirements were applied to select possible circRNA-associated-ceRNA networks that were likely involved in the occurrence and development of URSA. Moreover, the potential mechanisms via which circRNAs binding to hsa-miR-204-5p enhanced MFGE8 expression, thereby contributing to URSA were verified.

Our results showed that mRNA regulated URSA via multiple signaling pathways, including antigen processing and presentation, the Toll-like receptor signaling pathway, and Natural killer cell mediated cytotoxicity. Classically, activated macrophages in decidua are mainly involved in antigen processing and presentation (Li et al., 2021) which has been shown to be associated with URSA (Huang et al., 2021). Toll like receptors are a family of pattern recognition receptors, which play an essential role in maintaining innate host immunity and Th1/Th2 balance (Koga and Mor, 2010; Chen et al., 2016), thereby permitting and accepting implanted and growing semi allogenic embryos. Uterine NK (uNK) cells, which form an important part of the innate endometrial immune system, make up approximately 70% of all uterine lymphocytes during pregnancy and play a critical role in establishing and maintaining pregnancy. Numerous studies have investigated the role of uNK cells in adverse pregnancy outcomes, such as recurrent miscarriage (RM) or recurrent implantation failure (RIF), where many fertility clinics have provided immunotherapy to patient groups based on abnormal levels or activity of NK cells (Tenger et al., 2005; Perricone et al., 2006; Moffett and Shreeve, 2015; Mekinian et al., 2016; Plaçais et al., 2020). Using the PPI network, we identified ten hub genes, *CCL4*, *DDX58*, *CXCL10, CXCL9*, *MX1, CD44*, *RPS2, SOCS3*, *RPS3A,* and *CXCL11*. According to reports, these genes, which are mainly involved in inflammatory functions (Camacho-Arroyo et al., 2021; Jiang et al., 2021), and innate immunity (Nizyaeva et al., 2019), are essential for maintaining normal pregnancy.

Previous studies have indicated that ncRNAs are involved in several stages of implantation and contribute to the dysfunction of trophoblasts in pregnancy-related disorders (He et al., 2013; Chen H. et al., 2015). A study conducted by Dong et al., revealed that miRNAs that were abnormally and significantly expressed in villi and decidua, were involved in several biological processes, including the regulation of transcription and protein amino acid phosphorylation, while KEGG pathway analysis indicated that the predicted target genes were related to adherence junctions, apoptosis, and the T cell receptor signaling pathway (Dong et al., 2014). Another study revealed that the target genes of these miRNAs were closely associated with the mitogen-activated protein kinase (MAPK) pathway, as well as with the B cell receptor, and T cell receptor signaling pathways in the URSA development process (Tang et al., 2016). Similar to these results, the results of the current study indicated that 139 circRNAs were significantly differentially expressed in human decidua, compared to NEP, and that these were primarily involved in Rap1 signaling pathways, the TGF-beta signaling pathway, and the tight junction and T cell receptor signaling pathways. Rap1 signaling pathway is implicated in several basic cellular functions, including cell-cell interactions, cell–matrix adhesion, proliferation, and regulation of cellular polarity during fetal development. Magdalena et al., highlighted the key role of Rap1 signaling in maintaining epithelial and endothelial cell junction integrity that helps maintain fetus viability (Chrzanowska-Wodnicka et al., 2015). TGF-beta, which is important in ensuring maternal support for embryo development, modulates the extent of decidua invasion (Jones et al., 2006), which finding was substantiated by our circRNA pathway analysis. Both circRNAs and miRNAs were enriched in junction related pathways, including tight junction, gap junction and focal junction, which are essential for normal pregnancy (Fukuda and Sugihara, 2008; 2012), and in T cell adhesion to extracellular matrix proteins, which have been described as determinants of successful or failing pregnancy (Chaiworapongsa et al., 2002). Tang et al., suggested that downregulation of miR-181d (Tang et al., 2016) which contains relevant binding sites for upregulated circRNAs, may contribute to the inhibition of adhesion and cell proliferation in RSA NK cells (Qian et al., 2017). But, hsa-miR-141-3p which was top 10 of differentially expressed microRNAs was low expression in the present study, while high expression in NK cells isolated from the decidua of URSA (Li and Li, 2016). A previous study in mouse showed that embryo implantation sites were significantly decreased (Liu et al., 2013), following uteri horn injection of inhibitors or mimic of miR-141. The inconsistency between the results of the present study and previous studies may be due to the existence of a balance between the differentiation and apoptosis functions performed by hsa-miR-141-3p. Considered together, our findings further demonstrated the vital role of ncRNAs in URSA development.

The functions of circRNAs include sponging miRNAs, competing with endogenous RNAs, regulating gene transcription, and interacting with RNA-binding proteins (RBP) (Hansen et al., 2013). In particular, our results confirmed that the upregulated circRNA (has_circ_0004558, novel_circ_0006147, etc.) acted as ceRNAs of hsa-miR-204-5p, targeting MFGE8 in patients with URSA. MFGE8 is a glycoprotein which has been widely studied by many engaged in physiological and pathological research, especially regarding its role in the immune system (Matsuda et al., 2011; Oba et al., 2011). Previous studies have suggested that it participates in the implantation process, because it was found upregulated during the window of implantation (Mirkin et al., 2005; Franchi et al., 2011; Schmitz et al., 2014). On the other hand, MFGE8 is also known to be involved in the inflammatory process (Komura et al., 2009), and is regulated by TNF-α in the human endometrium (Yu et al., 2014). It has been reported that overexpression of MFGE8 may impair physiological processes (Yamamoto et al., 2014; Zhao et al., 2015). In keeping with this notion, Schmitz et al*.*(Schmitz et al., 2017) reported that MFGE8 expression in the glandular epithelium of patients with endometriosis and infertility was significantly increased, when compared to that of healthy fertile patients. The present study also demonstrated that the expression levels of MFGE8 mRNA and protein were increased in women with URSA. In summary, these results indicated that circRNAs played a crucial role in the occurrence of URSA, and that the sponging function of miRNAs may be the main pathway involved. Our study was affected by several limitations which should be acknowledged. Firstly, the small cohort studied was a major limitation that prevented us from drawing clear conclusions from our results. Secondly, the present study was unable to substantiate direct interaction between ceRNAs.

Collectively, we found that the expression levels of circRNAs, miRNAs, and mRNAs in the URSA differed significantly from those in the NEP group. In addition, our results enabled us to propose probable pathways via which circRNAs function in URSA. Furthermore, circRNA-associated ceRNAs were constructed and MFGE8 was identified as a critical component of the circRNA-miRNA network. These novel networks may lead to the discovery of potential biomarkers or therapeutic targets in URSA.

## Data Availability

The original contributions presented in the study are publicly available. This data can be found here: https://dataview.ncbi.nlm.nih.gov/object/PRJNA819201 and https://dataview.ncbi.nlm.nih.gov/object/PRJNA819210.
