# Supplementary material for: Comprehensive Analysis of circRNAs, miRNAs, and mRNAs Expression Profiles and ceRNA Networks in Decidua of Unexplained Recurrent Spontaneous Abortion
Source: Front Genet. 2022 May 31;13:858641. doi: 10.3389/fgene.2022.858641 (PMC9194479; doi:10.3389/fgene.2022.858641)
Supplement: Supplementary file 1 [file DataSheet1.docx]

Supplementary Material

# Comprehensive analysis of circRNAs, miRNAs and mRNAs expression profiles and ceRNA networks in decidua of unexplained recurrent spontaneous abortion

Xiaohua Liu^1, 2*^, Jiabao Wu^1, 2*^, Hua Nie^1, 2^, Xiaoli Zhu^1, 4^, Ge Song^1, 4^, Lu Han ^1, 2△^, Weibing Qin^1, 2△^

1 NHC Key Laboratory of Male Reproduction and Genetics, Guangzhou 510600, P. R. China;

2 Department of Center Laboratory, Guangdong Provincial Reproductive Science Institute, Guangzhou 510600, P. R. China;

3 Human Sperm Bank of Guangdong Province, Guangzhou 510600, P.R. China;

4 Reproductive Medicine Center, Guangdong Provincial Reproductive Science Institute, Guangzhou 510060, China.

*Xiaohua Liu and Jiabao Wu contributed equally to this work.

# Supplementary Figures and Tables


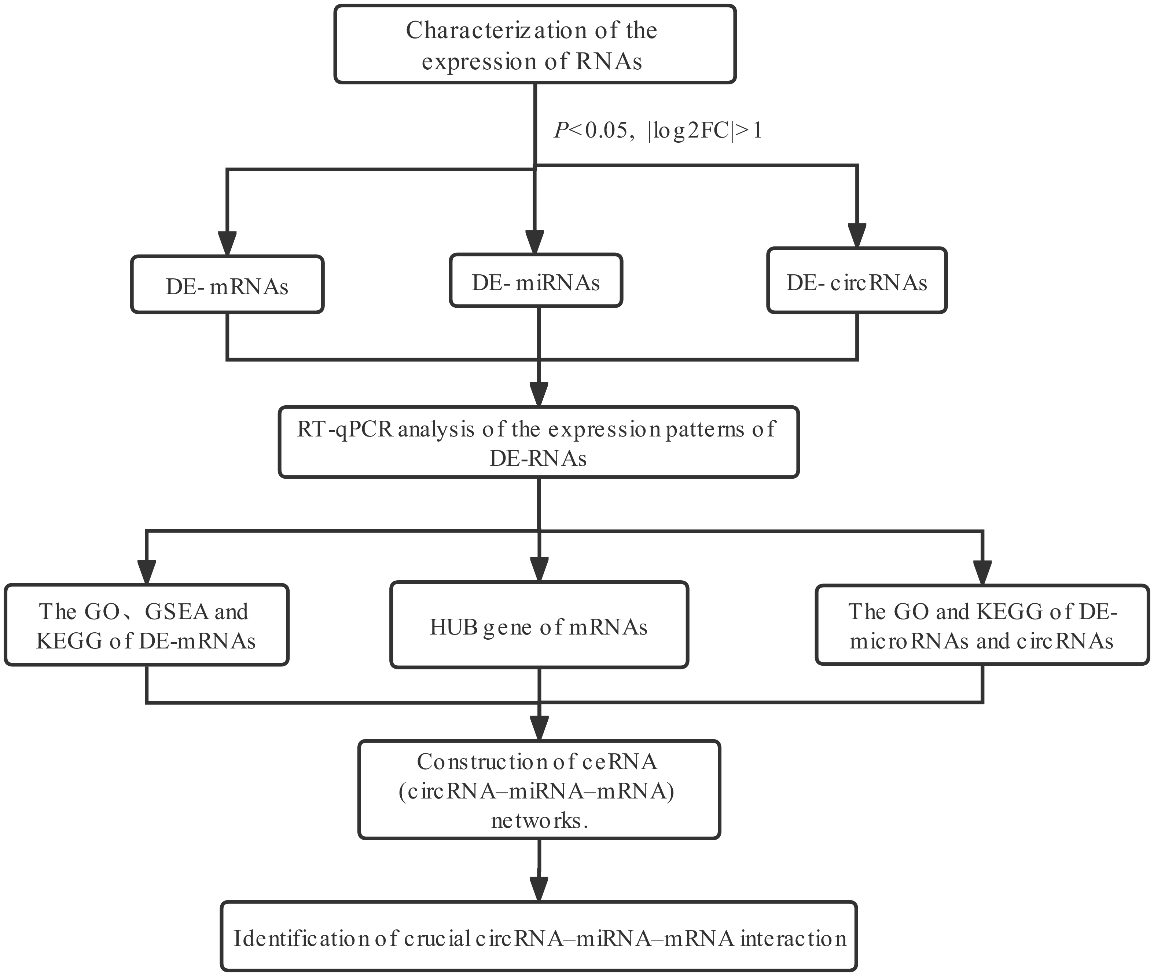


### Figure S1. ceRNA network construction flow chart.

log2FC: log2 fold change, DE: differentially expressed, ceRNA: competitive endogenous RNA, circRNA: circular RNA, miRNA: microRNA, mRNA: messenger RNA

### Table S1 Primers used in real-time PCRs for detecting RNAs expression.

| Gene name | Primer sequence (5’–3’) |
| --- | --- |
| hsa_circ_0004558 | ACCAGTGATAAGAAGGCCAATG  TGGGACAGTAAGTGAAGGCT |
| hsa_circ_0003653 | ACTGTGACCCCAGAAAATGG  TTGGGCAGTCAACACTTCAG |
| hsa_circ_0003234 | GGATCTGGCCGAGAACATAA  TTCCCTCTGCCACCTCATAC |
| hsa-miR-204-5p | TTCCCTTTGTCATCC TATGCCT  mRQ 3' primer* |
| hsa-miR-200c-3p | TAATACTGCCGGGTAATGATGGA  mRQ 3' primer* |
| hsa-miR-141-3p | TaacacTgTcTggTaaagaTgg  mRQ 3' primer* |
| CCX4 | TGTCCTGTCTCTCCTCATGC  TCGCGGTGTAAGAAAAGCAG |
| DDX58 | CTAAGGGGATGATGGCAGGT  GGGCCAGTTTTCCTTGTCTG |
| CXXL10 | GCAAGCCAATTTTGTCCACG  TGATGGCCTTCGATTCTGGA |
| ITGVA | Ggaaacccaatgaaggctgg  tggtgcacactgaaacgaag |
| TP53BP2 | Aagaattcctcggccactca  ttagaggccttggtgcgtta |
| MFGE8 | ACCTGTTTGAGACCCCTGTG  GGTTCCAGCTGAAGAGATGC |
| β-Actin | CGGGAAATCGTGCGTGAC  GGAAGGAAGGCTGGAAGAGTG |
| U6 | CTCGCTTCGGCAGCACA  AACGCTTCACGAATTTGCGT |

*mRQ 3' primer was provided by the Mir-X™ miRNA First-Strand Synthesis and SYBR® qRT-PCR kit (Takara).

### Table S2 circRNA-related ceRNA networks are most likely to participate in the pathogenesis of URSA

| CircRNA | Log2 Fold Change | *p* value | miRNA | Log2 Fold Change | Corrected  *p* value | Transcript_id | Gene_ID | Log2 Fold Change | Corrected  *p* value |
| --- | --- | --- | --- | --- | --- | --- | --- | --- | --- |
| hsa_circ_0004558 | 3.4613 | 0.0362 | hsa-miR-204-5p | -0.99985 | 0.00812 | ENST00000268150 | ENSG00000140545 | 1.393012 | 0.007579 |
| novel_circ_0006147 | 2.3171 | 0.02043 |  | -0.99985 | 0.00812 |  |  |  |  |
| hsa_circ_0008546 | 3.926 | 0.014388 |  | -0.99985 | 0.00812 |  |  |  |  |
| hsa_circ_0008797 | 2.6336 | 0.036767 |  | -0.99985 | 0.00812 |  |  |  |  |
| hsa_circ_0001402 | 0.95915 | 0.046042 |  | -0.99985 | 0.00812 |  |  |  |  |
| hsa_circ_0001997 | 3.4228 | 0.039368 |  | -0.99985 | 0.00812 |  |  |  |  |
| hsa_circ_0004596 | 3.3265 | 0.048545 |  | -0.99985 | 0.00812 |  |  |  |  |
| hsa_circ_0005630 | 1.7404 | 0.009199 |  | -0.99985 | 0.00812 |  |  |  |  |
| novel_circ_0008332 | 3.6976 | 9.85E-05 |  | -0.99985 | 0.00812 |  |  |  |  |
| hsa_circ_0003611 | 1.3359 | 0.036784 |  | -0.99985 | 0.00812 |  |  |  |  |
| hsa_circ_0060158 | 3.4507 | 0.037569 |  | -0.99985 | 0.00812 |  |  |  |  |
| hsa_circ_0056891 | 3.3828 | 0.042146 |  | -0.99985 | 0.00812 |  |  |  |  |
| hsa_circ_0025830 | 3.8398 | 0.017916 |  | -0.99985 | 0.00812 |  |  |  |  |
